# Cytotoxicity of VEGF_121_/rGel on vascular endothelial cells resulting in inhibition of angiogenesis is mediated via VEGFR-2

**DOI:** 10.1186/1471-2407-11-358

**Published:** 2011-08-17

**Authors:** Khalid A Mohamedali, Sophia Ran, Candelaria Gomez-Manzano, Latha Ramdas, Jing Xu, Sehoon Kim, Lawrence H Cheung, Walter N Hittelman, Wei Zhang, Johannes Waltenberger, Philip E Thorpe, Michael G Rosenblum

**Affiliations:** 1 Departments of Experimental Therapeutics, The University of Texas M. D. Anderson Cancer Center, Houston, TX, USA; 2 Departments of Neuro-Oncology, The University of Texas M. D. Anderson Cancer Center, Houston, TX, USA; 3 Departments of Experimental Radiation Oncology, The University of Texas M. D. Anderson Cancer Center, Houston, TX, USA; 4 Departments of Pathology, The University of Texas M. D. Anderson Cancer Center, Houston, TX, USA; 5 Department of Medical Microbiology and Immunology, Southern Illinois University, School of Medicine, Springfield, IL, USA; 6 Department of Cardiology and Angiology, University Hospital Münster, Münster, Germany; 7 Simmons Comprehensive Cancer Center, The University of Texas Southwestern Medical Center, Dallas, TX, USA; 8 The University of Texas Southwestern Medical Center, Dallas, TX, USA; 9 GlycoFi/Merck & Co., Inc., Lebanon, NH, USA

## Abstract

**Background:**

The fusion protein VEGF_121_/rGel composed of the growth factor VEGF_121 _and the plant toxin gelonin targets the tumor neovasculature and exerts impressive anti-vascular effects. We have previously shown that VEGF_121_/rGel is cytotoxic to endothelial cells overexpressing VEGFR-2 but not to endothelial cells overexpressing VEGFR-1. In this study, we examined the basis for the specific toxicity of this construct and assessed its intracellular effects *in vitro *and *in vivo*.

**Methods:**

We investigated the binding, cytotoxicity and internalization profile of VEGF_121_/rGel on endothelial cells expressing VEGFR-1 or VEGFR-2, identified its effects on angiogenesis models *in vitro *and *ex vivo*, and explored its intracellular effects on a number of molecular pathways using microarray analysis.

**Results:**

Incubation of PAE/VEGFR-2 and PAE/VEGFR-1 cells with ^125^I-VEGF_121_/rGel demonstrated binding specificity that was competed with unlabeled VEGF_121_/rGel but not with unlabeled gelonin. Assessment of the effect of VEGF_121_/rGel on blocking tube formation *in vitro *revealed a 100-fold difference in IC_50 _levels between PAE/VEGFR-2 (1 nM) and PAE/VEGFR-1 (100 nM) cells. VEGF_121_/rGel entered PAE/VEGFR-2 cells within one hour of treatment but was not detected in PAE/VEGFR-1 cells up to 24 hours after treatment. In vascularization studies using chicken chorioallantoic membranes, 1 nM VEGF_121_/rGel completely inhibited bFGF-stimulated neovascular growth. The cytotoxic effects of VEGF_121_/rGel were not apoptotic since treated cells were TUNEL-negative with no evidence of PARP cleavage or alteration in the protein levels of select apoptotic markers. Microarray analysis of VEGF_121_/rGel-treated HUVECs revealed the upregulation of a unique "fingerprint" profile of 22 genes that control cell adhesion, apoptosis, transcription regulation, chemotaxis, and inflammatory response.

**Conclusions:**

Taken together, these data confirm the selectivity of VEGF_121_/rGel for VEGFR-2-overexpressing endothelial cells and represent the first analysis of genes governing intoxication of mammalian endothelial cells by a gelonin-based targeted therapeutic agent.

## Background

Continuing investigations into the biology of tumor-stromal interactions have identified a number of pathways and events critical to the development and maintenance of tumors and their metastatic spread. Tumor neovascularization is a critical, robust process dependent on the interplay between numerous soluble cytokines, growth factors and their receptors. Targeted therapy focusing on the tumor neovascularization process appears to be a promising approach in this regard [1]. The VEGF-A family of cytokines and their cognate receptors have been identified as key mediators of angiogenesis and endothelial cell proliferation, migration and survival [2-6], and play a central role in the organization of solid tumor vasculature [7,8].

The smallest of the VEGF isoforms, VEGF_121 _binds to two receptors designated VEGFR-1 (Flt-1/FLT-1) and VEGFR-2 (Flk-1/KDR), both of which are over-expressed on the endothelium of tumor vasculature but virtually undetectable in the vascular endothelium of adjacent normal tissues. We have previously characterized a novel fusion construct of VEGF_121 _and the plant toxin Gelonin (rGel). Gelonin is a 28.5 kDa single-chain protein belonging to the family of Type 1 plant Ribosome-Inactivating Proteins (RIPs) that can hydrolyze the glycosidic bond of a highly conserved adenosine residue in the largest RNA in the 28S ribosome, resulting in irreversible inhibition of protein synthesis. *In vivo*, VEGF_121_/rGel targets and destroys tumor neovasculature in solid tumors [9,10], reduces breast cancer metastatic spread and dramatically reduces neovascularization of pulmonary breast metastases [11], prevents tumor growth in bone in osteolytic and osteoblastic bone metastasis models [12,13], and blocks retinal and choroidal neovascularization in studies of experimental ocular neovascular disease [14]. The binding of VEGF_121_/rGel to both VEGFR-1 and VEGFR-2 has been demonstrated *in vivo *using non-invasive bioluminescence imaging (BLI), magnetic resonance imaging (MRI) and positron-emission tomography (PET) [15]. Thus, VEGF_121_/rGel appears to be a promising candidate for targeting its cognate receptors in various disease states.

Interestingly, VEGF_121_/rGel demonstrates targeted toxicity *in vitro *to endothelial cells which over-express VEGFR-2 (IC_50 _= 0.5 - 1 nM) but not to cells which over-express VEGFR-1 (IC_50 _= 300 nM) compared to gelonin alone (IC_50 _= 300 nM) [10]. This is surprising since VEGF_121 _binds to both receptors with affinity in the picomolar range [16]. There are several possibilities that may account for this difference in toxicity: (a) the binding affinity of VEGF_121_/rGel to VEGFR-1 may be reduced, (b) binding affinity is not affected but the rate of internalization of VEGF_121_/rGel bound to VEGFR-1 is reduced compared to VEGFR-2 and (c) different access to the ribosomal machinery following cell entry due to being trapped in the endosomal compartment. In addition, while the molecular effects of VEGF_121_-treatment of endothelial cells have been studied [17], the effects of VEGF_121_/rGel on endothelial cells have yet to be elucidated. This information is critical in the context of *in vivo *targeting because of the potential role that stimulation by VEGF_121 _can have on cell survival and rGel-mediated toxicity. For example, VEGF_121 _may activate particular signal transduction pathways early in the process that can result in increased toxicity of the rGel component even prior to complete inhibition of protein synthesis. The biochemical process of drug action, and its off-target effects can best be studied under controlled conditions *in vitro*. In this report, we focus on understanding the mechanism of action of VEGF_121_/rGel on endothelial cells by determining its binding profile to VEGFR-1 and VEGFR-2, identifying its effects on angiogenesis models *in vitro *and *ex vivo*, and exploring its intracellular effects on a number of molecular pathways using microarray analysis.

## Methods

### Cell Culture

Porcine aortic endothelial (PAE) cells transfected with the human VEGFR-2 (PAE/VEGFR-2) or the human VEGFR-1 (PAE/VEGFR-1) have been used as *in vitro *models of angiogenesis [18]. The number of R-2 and R-1 receptor sites on these cells lines have been previously determined at 150,000 and 50,000 per cell, respectively [19]. Human umbilical vein endothelial cells (HUVECs) were maintained in EBM medium (Cambrex, East Rutherford, NJ).

### Purification of VEGF_121_/rGel

Construction and purification of VEGF_121_/rGel was essentially as described [10]. VEGF_121_/rGel was concentrated and stored in sterile PBS at -20°C.

### Western Blot Analysis

Whole cell extracts of HUVECs, PAE/VEGFR-2 and PAE/VEGFR-1 cells were prepared as described [11]. Western blotting was performed using antibodies for actin (loading control), VEGFR-2, p-VEGFR-2 (p-KDR), E-selectin, and various apoptotis markers.

### Cell Surface Binding of Radiolabeled VEGF_121_/rGel to PAE/VEGFR-2 and PAE/VEGFR-1 cells

VEGF_121_/rGel was radiolabeled with 1mCi of NaI^125 ^using Chloramine T [20] for a specific activity of 602 Ci/mMol. Equivalent numbers of cells were grown overnight in 24-well plates and cell surface binding assays were performed as described previously [21]. Briefly, non-specific binding sites were blocked for 30 minutes with PBS/0.2% gelatin followed by incubation for 4 hours at 4°C with ^125^I-VEGF_121_/rGel (10 nM) in PBS/0.2% gelatin solution. For competition experiments, cold VEGF_121_/rGel or gelonin (400 nM) were pre-mixed with ^125^I-VEGF_121_/rGel. Cells were washed four times with PBS/0.2% gelatin solution, detached and bound cpm was measured.

### Cytoxicity and Internalization of VEGF_121_/rGel and rGel

Cytotoxicity of VEGF_121_/rGel and rGel against log phase PAE/VEGFR-2 cells was performed over 72 hours as described for PAE/KDR and PAE/FLT-1 cells [10]. To assess if the activity of VEGF_121_/rGel was affected by the exposure time to endothelial cells, log-phase PAE/VEGFR-2 cells were treated with VEGF_121_/rGel and media containing the cytotoxic agent was removed at varying time-points and replaced with fresh media. For internalization, cells were treated with 4 μg/ml (48 nM) VEGF_121_/rGel at the timepoints indicated, then washed with Glycine buffer (500 mM NaCl, 0.1 M glycine, pH 2.5) to remove cell surface-bound VEGF_121_/rGel. Cells were incubated with a rabbit anti-gelonin polyclonal antibody (1:200) followed by a FITC-conjugated anti-rabbit secondary antibody (1:80). Nuclei were stained with propidium iodide (1 μg/ml) in PBS. The slides were mounted with DABCO reagent and visualized under fluorescence (Nikon Eclipse TS1000) and confocal (Zeiss LSM 510) microscopes.

### TUNEL Assay

Log phase PAE/VEGFR-2 and PAE/VEGFR-1 cells (2000 cells/well) were treated with 1 nM VEGF_121_/rGel for 72, 48 and 24-hours. The cells were then processed and analyzed for TUNEL as described by the manufacturer of the reagent (Roche Diagnostics, Indianapolis, IN).

### Endothelial Cell Tube Formation Assay

PAE/VEGFR-2 and PAE/VEGFR-1 cells plates on Matrigel were treated with 0.01 - 100 nM VEGF_121_/rGel or rGel, in triplicate, for 24 h. Inhibition of tube formation was assessed by counting the number of tubes formed per well under bright field microscopy. The ability of VEGF_121_/rGel to inhibit tube formation as a function of incubation time before plating on Matrigel was studied by incubating PAE/VEGFR-2 cells at the IC_50 _dose (1 nM) for different periods up to 24 h. Cells were detached and plated in 96-well Matrigel-coated plates under the conditions described above and the tubes in each well were counted.

### Angiogenesis Assessment in Chicken Chorioallantoic Membranes

Chorioallantoic membrane (CAM) experiments using fertilized chicken eggs (SPAFAS; Charles River Laboratories, Wilmington, MA) were performed as described [22]. Experiments were performed twice per treatment, with 6 to 10 embryos per condition in every experiment. Each CAM was locally treated with filter disks saturated with a solution containing bFGF (50 ng/disk) and VEGF_121_/rGel (1 or 10 nM), rGel (1 or 10 nM), or buffer (PBS). The filter was placed on the CAM in a region with the lowest density of blood vessels and, as a reference, in the vicinity of a large vessel. Angiogenesis was documented photographically 3 days after treatment; images were captured using an Olympus stereomicroscope (SZ x12) and Spot Basic software (Diagnostic Instruments, Inc.). The relative vascular density was determined by measuring the area occupied by blood vessels [23] using the public domain NIH Image program (available on the Internet at http://rsb.info.nih.gov/nih-image/). The numbers of blood vessel branch points were independently and blindly quantified by two researchers (C.G-M. and J.X.) and compared with the numbers in the treatment controls [22].

### RNA Extraction, Gene Expression Analysis, and RT-PCR Correlative Analysis

HUVECs and PAE/VEGFR-2 cells were treated with their respective IC_50 _VEGF_121_/rGel doses for 24 h. Control cells were treated with PBS. Total RNA was extracted and analyzed for integrity as described [11]. HUVEC RNA was amplified and labeled using Cy3- and Cy5-dCTP in the reverse transcription reaction. Duplicate experiments were conducted by dye swapping. The labeled samples were hybridized to a cDNA array of 2304 sequence-verified clones in duplicate printed by the Cancer Genomics Core Laboratory (MDACC). The array included 4800 genes involved in signal transduction, stress response, cell cycle control, hypoxia, and metastatic spread. Differentially expressed genes were identified on the basis of a cutoff value of the *T *value. Generally, a cutoff value of |3| is considered statistically significant. Genes that showed fold changes greater than |2| in at least 3 of 4 arrays were identified, and the average fold change was determined. Microarray data were verified by performing RT-PCR analysis on the genes that showed the highest level of induction, namely E-selectin (SELE), cytokine A2 (SCYA2, MCP-1), tumor necrosis factor alpha induced protein 3 (TNFAIP3) and NF-κB inhibitor alpha (NF-κBIα). Primers were designed on the basis of the accession numbers from the microarray and confirmation of homology using BLAST (NCBI). Induction of E-selectin in PAE/VEGFR-2 cells was also verified by RT-PCR. GAPDH primers were used as controls.

## Results

### VEGF_121_/rGel binds to both VEGFR-1 and VEGFR-2

**Figure 1:**
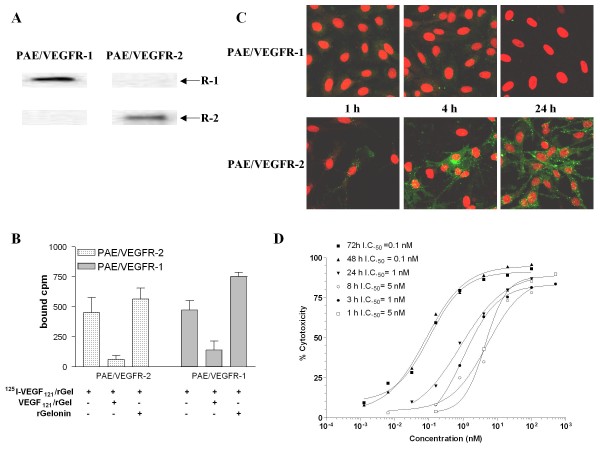
**VEGF_121_/rGel binds to both VEGFR-1 and VEGFR-2 but is cytotoxic only to VEGFR-2-expressing endothelial cells**. *A*, Expression of both receptors on their respective cell-lines was confirmed. *B*, Receptor-specific binding of radiolabeled VEGF_121_/rGel. Binding was reduced with unlabeled VEGF_121_/rGel but not by unlabeled gelonin. *C*, VEGF_121_/rGel enters PAE/VEGFR-2 cells within one hour of treatment. However, PAE/VEGFR-1 cells did not internalize VEGF_121_/rGel even after 24 hours of incubation with VEGF_121_/rGel. *D*, Effect of exposure time of VEGF_121_/rGel on PAE/VEGFR-2 cells on cytotoxicity.

VEGF_121 _has been previously shown to bind to both VEGFR-1 and VEGFR-2 with similar affinity [16]. Cells over-expressing VEGFR-2 are nearly 600-fold more sensitive to VEGF_121_/rGel than cells expressing VEGFR-1 [10] despite just a 3-fold higher receptor number on the cell surface. We investigated the relative binding of VEGF_121_/rGel to PAE cells expressing each of the receptors. Expression of the two receptors was confirmed by Western blot (Figure 1A). Total binding of ^125^I-VEGF_121_/rGel to both PAE/VEGFR-1 and PAE/VEGFR-2 cells was nearly identical and was effectively competed by unlabeled VEGF_121_/rGel but not by unlabeled gelonin, indicating that binding of VEGF_121_/rGel was mediated by the VEGF_121 _component of the construct and, therefore, the cytotoxic effects were VEGF receptor-mediated (Figure 1B). Interestingly, the presence of free gelonin seemed to slightly increase binding of ^125^I-VEGF_121_/rGel to both cells. The reason for this is unclear but may be due to gelonin blocking non-specific sites that may otherwise bind VEGF_121_/rGel.

### VEGF_121_/rGel is internalized into PAE/VEGFR-2 cells but not into PAE/VEGFR-1 cells

Because VEGF_121_/rGel appears to bind VEGFR-1, we examined the role of internalization to explain the lack of cytotoxicity of VEGF_121_/rGel on PAE/VEGFR-1 cells. After incubation of cells with VEGF_121_/rGel, the cell surface was stripped to probe only for internalized protein. VEGF_121_/rGel was detected in PAE/VEGFR-2 cells within 1 hour of treatment with the immunofluorescence signal progressively increasing by 24 hours (Figure 1C). No VEGF_121_/rGel was detected in PAE/VEGFR-1 cells up to 24 hours after treatment with the fusion toxin. Because no internalization is observed into PAE/VEGFR-1 cells at both short and long time points, it is unlikely that differences in receptor recycling rates, if any, in the two receptor-transfected cells contribute to these observations. Treatment of cells with the same concentration of gelonin also showed no internalization (see Additional file 1), confirming that entry of VEGF_121_/rGel into PAE cells occurred almost exclusively via VEGFR-2.

### VEGF_121_/rGel cytotoxicity on endothelial cells correlates with exposure time

Because VEGF_121_/rGel internalized into PAE/VEGFR-2 cells within one hour of incubation, we studied the cytotoxic effect of VEGF_121_/rGel as a function of exposure time of this agent on endothelial cells. PAE/VEGFR-2 cells were treated with VEGF_121_/rGel from 1-72 hours and the cytotoxic effect was assessed at the end of the 72-hour period. VEGF_121_/rGel demonstrated targeted toxicity even after a one-hour exposure, with an IC_50 _of 5 nM. The maximal cytotoxic effect of VEGF_121_/rGel on PAE/VEGFR-2 cells was observed at 48 and 72 hours (IC_50 _= 0.1 nM) (Figure 1D). The cytotoxic effect of VEGF_121_/rGel on PAE/VEGFR-1 cells was similarly affected as a function of exposure duration (see Additional file 2) in that longer exposure times resulted in higher cytoxicity with the maximum cytotoxic effect occurring at 72 h (IC_50 _= 100 nM).

### VEGF_121_/rGel treatment activates VEGFR-2 downstream signaling

**Figure 2:**
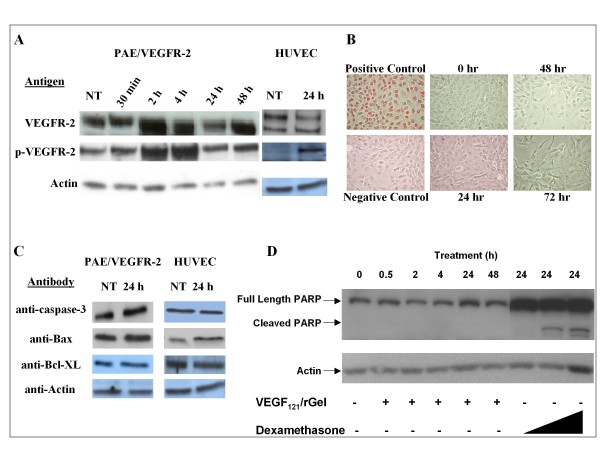
**Cytotoxicity of VEGF_121_/rGel to PAE/VEGFR-2 cells does not result in apoptosis**. *A*, Decreased VEGFR-2 and increased p-VEGFR-2 levels in PAE/VEGFR-2 cells and HUVECs treated with VEGF_121_/rGel at IC_50 _doses for 24 h. Actin levels were used as a loading control. NT, not treated. *B*, TUNEL analysis of PAE/VEGFR-2 cells treated with 1 nM VEGF_121_/rGel for 24, 48 and 72 hours. Positive control cells were incubated with 1 mg/ml DNAse for 10 minutes at 37°C. *C*, Lack of effect of VEGF_121_/rGel on apoptotic markers in endothelial cells. Protein levels of Bax, Bcl-XL and caspase-3 remained unchanged, suggesting that the mechanism of cell death induced by the construct is not apoptotic. Levels of cytochrome C, caspase-6, and Bcl-2 were undetectable (data not shown). NT, not treated. *D*, PAE/VEGFR-2 cells treated with VEGF_121_/rGel did not demonstrate PARP cleavage, while cells exposed to dexamethasone (positive control) showed cleaved PARP.

Activation of downstream signaling by the receptor-fusion protein complex may be necessary to facilitate the internalization of the receptor/ligand complex, and may also increase the susceptibility of the cell to the toxic effects of rGel. We evaluated endogenous levels of phosphorylated VEGFR-2 (p-VEGFR-2) in endothelial cells that had been treated with VEGF_121_/rGel. PAE/VEGFR-2 cells expressed levels of p-VEGFR-2 that increased within 2 h after VEGF_121_/rGel treatment. The levels of p-VEGFR-2 peaked at 4 h and gradually decreased to endogenous levels by 24 h posttreatment (Figure 2A, left panel). Endogenous levels of total VEGFR-2 were also increased by 4 h and were reduced to pretreatment levels by 24 h. In contrast, endogenous levels of total VEGFR-2 in HUVECs decreased slightly at 24 h after treatment with VEGF_121_/rGel, whereas p-VEGFR-2 levels after 24 h were markedly upregulated compared with the levels of untreated cells (Figure 2A, right panel).

### Cytotoxic effects of VEGF_121_/rGel on endothelial cells are not mediated via apoptotic mechanisms

Nuclei of positive control PAE/VEGFR-2 cells showed intense TUNEL staining (Figure 2B). In contrast, no TUNEL staining was observed with PAE/VEGFR-2 cells exposed to VEGF_121_/rGel up to 72 hours, indicating that the mechanism of cytotoxicity of VEGF_121_/rGel did not involve apoptosis. To confirm this observation, we examined various key apoptotic signaling events using Western blot analysis. Levels of caspase-3 (full length pre-cursor), Bax (a pro-apoptotic protein), and Bcl-XL (an apoptosis inhibitor) were not found to be affected by VEGF_121_/rGel treatment (Figure 2C). In addition, the p11 and p20 subunits of activated/cleaved caspase-3 were not detected after treatment with the fusion construct. Levels of the pro-apoptotic molecules cytochrome C and caspase-6, as well as the anti-apoptotic protein Bcl-2 were undetectable before or after treatment (data not shown). PARP cleavage was tested on PAE/VEGFR-2 cells by treating cells with VEGF_121_/rGel or VEGF_121 _for periods ranging from 5 minutes to 48 hours. Western blot analysis of these cells by an anti-PARP antibody showed that neither VEGF_121_/rGel nor VEGF_121 _activated PARP-mediated apoptosis (Figure 2D; VEGF_121 _data in Additional file 3).

### VEGF_121_/rGel inhibits tube formation in VEGFR-2-expressing endothelial cells

**Figure 3:**
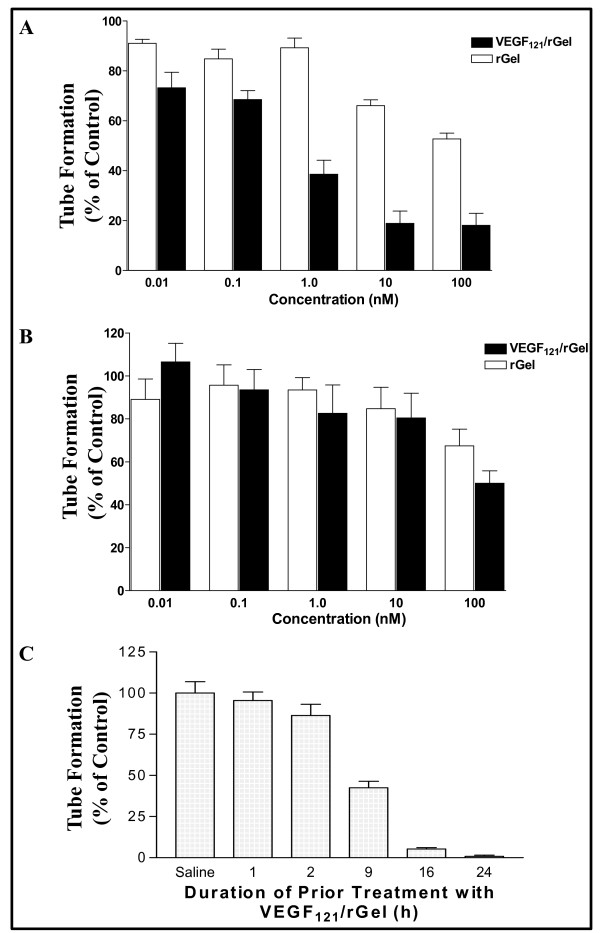
**VEGF_121_/rGel mediates inhibition of tube formation in PAE/VEGFR-2 cells**. *A*, One nM VEGF_121_/rGel was sufficient to inhibit tube formation by 50%, whereas the same degree of inhibition was seen with rGel only at 100 nM. *B*, Up to 100 nM VEGF_121_/rGel was needed to inhibit tube formation in PAE/VEGFR-1 cells, the same concentration as the untargeted gelonin toxin. *C*, Time-dependent inhibition of tube formation of PAE/VEGFR-2 cells by VEGF_121_/rGel. Incubation of PAE/VEGFR-2 cells with VEGF_121_/rGel for as little as 9 h was sufficient to abolish the ability of these cells to form tubes by 50%.

We next investigated the anti-angiogenic effect of VEGF_121_/rGel on tube formation of endothelial cells on Matrigel coated plates, a well established *in vitro *assay for angiogenesis. The addition of 1 nM VEGF_121_/rGel was found to significantly inhibit tube formation in PAE/VEGFR-2 cells compared to untreated cells, whereas rGel alone had little effect at this dose level (Figure 3A). rGel alone caused ~42% inhibition at only the highest concentration tested (100 nM). PAE/VEGFR-1 cells were not as sensitive to VEGF_121_/rGel as PAE/VEGFR-2 cells, requiring 100 nM VEGF_121_/rGel or rGel to inhibit tube formation by 50% (Figure 3B). To determine whether pre-treatment of PAE/VEGFR-2 cells with VEGF_121_/rGel affects tube formation, cells were treated with the IC_50 _dose of VEGF_121_/rGel for 1 to 24 h, washed with PBS, detached, added to Matrigel-coated plates in VEGF_121_/rGel-free medium, and incubated for an additional 24 h. Prior incubation of cells with VEGF_121_/rGel for 16 or 24 h was found to virtually abolish the formation of tube structures (Figure 3C).

### VEGF_121_/rGel inhibits angiogenesis in the CAM of chicken embryos

**Figure 4:**
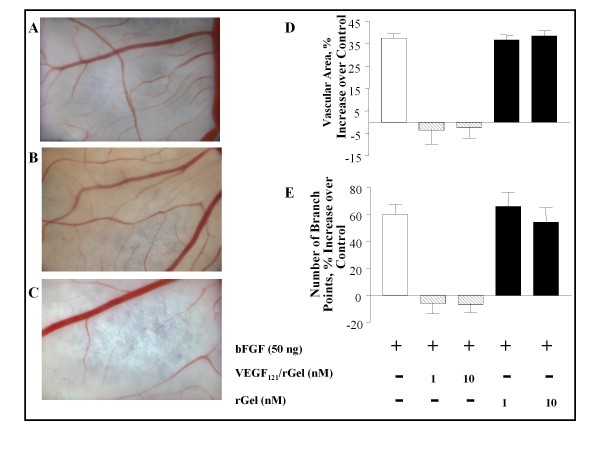
**VEGF_121_/rGel mediates inhibition of angiogenesis in chicken embryo CAMs via reduction of the vascular area and number of vascular branches**. *A*, Vasculature of a CAM after stimulation with bFGF alone. *B*, rGel had no effect on angiogenic stimulation of bFGF at either 1 nM or 10 nM. *C*, 1 nM VEGF_121_/rGel in the presence of 50 ng of bFGF inhibited angiogenesis. *D *and *E*, Quantitative evaluation of VEGF_121_/rGel-mediated inhibition of angiogenesis in the CAM model, normalized to CAMs treated with buffer (PBS; equal to 100%). *D*, VEGF_121_/rGel at both 1 and 10 nM decreased the vascular area. As expected, rGel alone had no effect. *E*, VEGF_121_/rGel decreased the number of newly sprouting vessels. VEGF_121_/rGel at a concentration of 1 nM dramatically affected the formation of the neovasculature, completely inhibiting bFGF-mediated stimulation of the neovasculature. As expected, rGel did not affect the number of newly sprouting vessels. Data shown represent the means ± standard deviations from replicated experiments. *, *P *< 0.001; *t*-test, double-sided.

We investigated the antiangiogenic effects of VEGF_121_/rGel *in vivo *using a chicken chorioallantoic membrane (CAM) model. The vascularized area in the CAMs treated with bFGF was about 35% higher than in those treated with PBS, the difference being highly significant (*P *< 0.001; *t*-test, double-sided; Figure 4A and 4D). This observation was consistent with the finding of more than a 60% increase in the number of newly-sprouted vessels in the bFGF-treated CAMs compared with the PBS-treated CAMs (*P *< 0.001; *t*-test, double-sided; Figure 4E). Incubation of CAMs with bFGF without or with 10 nM rGel resulted in normal angiogenic activity and the formation of an ordered neovasculature (Figure 4A and 4B) showing no impact of the rGel toxin. In contrast, treatment with 1 or 10 nM VEGF_121_/rGel resulted in considerable destruction of the neovasculature (Figure 4C) with complete inhibition of bFGF-stimulated angiogenesis (*P *< 0.001; *t*-test, double sided; Figure 4D and 4E). Many of the treated CAMs also appeared to be devoid of vessel infiltration. Interestingly, the number of branching points in the VEGF_121_/rGel-treated CAMs was similar to that in the PBS-treated CAMs (*P *> 0.5; *t*-test, double-sided; Figure 4E), suggesting that VEGF_121_/rGel mainly inhibits bFGF-mediated formation of newly sprouting branches from pre-existing vessels. As expected, the disks treated with bFGF in combination with rGel (at 1 or 10 nM) consistently showed extensive vascularization that was comparable to that found in those treated with bFGF alone (*P *> 0.5; *t*-test, double-sided).

### Microarray analysis of HUVECs treated indicates VEGF_121_/rGel upregulates genes involved in inflammation, chemotaxis and transcription regulation

**Figure 5:**
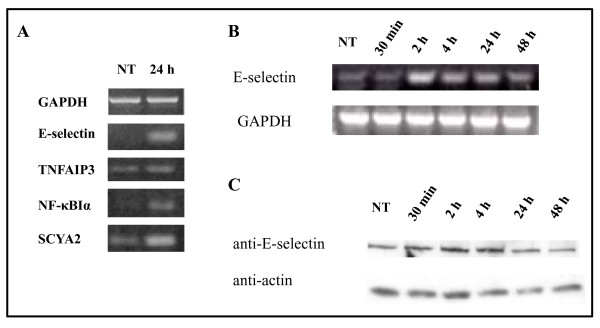
**Microarray and protein expression analysis of endothelial cells treated with VEGF_121_/rGel**. *A*, Validation of the microarray analysis by PCR is shown. Upregulation of genes for E-selectin, TNFAIP3, NF-κBIα and SCYA2 were validated by RT-PCR. GAPDH levels were assessed as a control. *B*, RNA from PAE/VEGFR-2 cells untreated or treated with VEGF_121_/rGel for the periods indicated were examined by PCR for upregulation of E-selectin. GAPDH primers were used as a control for loading. RNA levels of E-selectin were all upregulated in PAE/VEGFR-2 cells, as well as in HUVECs. *C*, Protein levels of E-selectin are only slightly upregulated compared to E-selectin RNA.

To further explore the intracellular effects of VEGF_121_/rGel and identify molecular pathways in endothelial cells that may influence cell survival and rGel-mediated toxicity, we treated HUVECs with saline or with an IC_50 _dose of VEGF_121_/rGel for 24 h. Rigorous microarray analysis resulted in selection of only those differentially expressed genes whose levels were elevated to at least 2 fold higher than the baseline values in repeated experiments. On this basis, 22 genes were found to be upregulated by treatment with VEGF_121_/rGel at 24 h (Table 1). In addition to upregulating select genes known to be induced by VEGF alone, treatment with VEGF_121_/rGel upregulated genes involved in inflammation, chemotaxis and transcription regulation. The genes with the highest levels of expression from four gene classifications were validated by RT-PCR analysis. When normalized for GAPDH, all four of the other PCR products were increased after treatment with VEGF_121_/rGel, thus validating the results observed in the original microarray (Figure 5A).

**Table 1:** HUVEC genes that increase following treatment with VEGF_121_/rGel for 24 hours, compared to untreated cells

Gene classification	Accession Number	Symbol	Gene	Mean fold change
Cell adhesion	H39560	SELE	E-selectin (endothelial adhesion molecule 1)*^a^*	94.6
	H07071	VCAM	Vascular cell adhesion molecule 1	4.9
	AA284668	PLAU	Plasminogen activator, urokinase	2.3

Apoptosis	AA476272	TNFAIP3	Tumor necrosis factor alpha-induced protein 3*^a^*	13.5
	H48706	BIRC3	baculoviral IAP repeat-containing 3	3.3

Transcription regulation	T99236	JUNB	jun B proto-oncogene	4.9
	W55872	NF-κBIα	nuclear factor of kappa light polypeptide gene enhancer in B-cells inhibitor, alpha*^a^*	4.8
	AA451716	NF-κB1	nuclear factor of kappa light polypeptide gene enhancer in B-cells 1 (p105)	2.3
	H45711	KLF4	Kruppel-like factor 4	2.3

Chemotaxis	AA425102	SCYA2	small inducible cytokine A2 (MCP-1)*^a^*	20.2
	H62985	SCYA4	small inducible cytokine A4 (MIP-1β)	5.8
	AA040170	SCYA7	small inducible cytokine A7 (MCP-3)	5.5
	T62491	CXCR4	chemokine (C-X-C motif), receptor 4 (fusin)	1.85

Structural organization	NM_004856	KNSL5	kinesin-like 5 (mitotic kinesin-like protein 1)	6.4
	AA479199	NID2	nidogen 2	3.1
	AA453105	H2AFL	H2A histone family, member L	2.5

Inflammatory response	W69211	SCYA11	small inducible cytokine A11 (Cys-Cys)	8.4
	NM_001964	EGR1	(eotaxin) early growth response 1	3.9
	NM_000963	PTGS2	prostaglandin-endoperoxide synthase 2 (COX-2)	3.3
	AA148736	SCD4	syndecan 4 (amphiglycan, ryudocan)	3.2

Signaling	W65461	DUSP5	dual specificity phosphatase 5 (MKP-1)	2.7

Metabolic	AA011215	SAT	spermidine/spermine N1-acetyltransferase	2.1

*^a ^*Confirmed by RT-PCR at 4 and 24 h posttreatment

Surprisingly, we did not observe E-selectin protein expression in HUVECs in control and 24 h - treated cells (data not shown). However, induction of E-selectin mRNA was observed (see Additional file 4) suggesting that the ribosomal machinery had been effectively inhibited. Because PAE/VEGFR-2 cells have been used as *in vitro *models for endothelial cells in the tumor neovasculature, we investigated the effect of VEGF_121_/rGel on gene induction and protein expression in these cells. PAE/VEGFR-2 cells were treated with saline or the IC_50 _dose of VEGF_121_/rGel for up to 48 h. PCR analysis for E-selectin confirmed the increase in message within 2 h after treatment of cells with VEGF_121_/rGel (Figure 5B). In addition, Western blot analysis demonstrated a slight increase in E-selectin protein expression, although the increase in cellular protein levels was modest compared with the observed increase in message, and was obvious only up to 4 h after treatment (Figure 5C). MKP-1 RNA levels were upregulated 2.7-fold in HUVECs (Table 1). Western blots against MKP-1 and ERK2, previously shown to be upregulated by MKP-1 in HUVECs following injury, also showed no change in protein expression (see Additional file 5).

## Discussion

Tumor neovascularization is highly dependent upon numerous cytokines and signaling events critical for the growth and organization of the vascular tree. A number of agents targeting tumor neovascularization and which interfere with one or several steps in this robust process have demonstrated significant clinical efficacy and have received FDA approval [24]. These include agents which block angiogenesis signaling events by inhibiting various growth factor receptor kinases [25]; interfere with VEGF physical interaction with its receptors such as anti-VEGF antibodies (bevacizumab and ranibizumab) and anti-receptor antibodies (IMC-1121B and DC101) [26,27]; and strategies that trap growth factor ligands (VEGF-Trap) [28]. These have all shown antitumor efficacy alone and in combination with conventional antitumor modalities [29,30].

VEGF-A has been shown to play an important role in tube formation of endothelial cells *in vitro *[31] and in angiogenesis [32]. In the present study, the effect of VEGF_121_/rGel on tube formation of endothelial cells on Matrigel-coated plates was striking in that cells overexpressing VEGFR-2, but not cells overexpressing VEGFR-1, were affected. This result is consistent with our findings that VEGF_121_/rGel is cytotoxic only to VEGFR-2-expressing endothelial cells [10] and is internalized only into endothelial cells that express VEGFR-2 but not VEGFR-1 (this study). The inhibition by VEGF_121_/rGel of tube formation *in vitro *translates well to inhibition of both vascular endothelial growth and neovasculature *in vivo *in the CAM membrane assays. The CAM assay also demonstrated that treatment with VEGF_121_/rGel did not affect mature vessels. This critical finding supports our hypothesis that VEGF_121_/rGel does not affect mature vessels in either normal tissues or tumors since both VEGFR-1 and VEGFR-2 are over-expressed on the endothelium of tumor neovasculature [33-36] but are almost undetectable in the vascular endothelium of adjacent normal tissues and in mature tumor vessels. Therefore, small, newly vascularizing tumors and metastases may be the lesions most responsive to therapy with this agent.

The lack of internalization of VEGF_121_/rGel into PAE/VEGFR-1 cells explains the difference in cytotoxicity compared to PAE/VEGFR-2. This also supports the hypothesis that VEGFR-1 is a decoy receptor, at least on endothelial cells, as it demonstrates weak tyrosine phosphorylation upon VEGF stimulation [34]. However, we have demonstrated that mouse monocytes internalize VEGF_121_/rGel via VEGFR-1 [12], suggesting that other factors may influence VEGFR-1 receptor activity such as cell type, total receptor number and dimerization partner.

While the mechanism of rGel itself is to target the ribosomal machinery, the extent to which translation is inhibited will affect downstream cellular responses, such as other mechanisms of cell death. Information about these mechanisms may reveal additional pathways that can be targeted in combination with the fusion toxin to achieve optimal efficacy. Our study demonstrates that the cytotoxic effect of VEGF_121_/rGel on VEGFR-2-overexpressing endothelial cells is not due to programmed cell death (apoptosis). Previous studies of a gelonin-based immunotoxin targeting tumor cells showed that intoxicated cells did not appear to display apoptotic characteristics [37]. In contrast, gelonin coupled to BlyS induced apoptosis in B cells [38] strongly supporting the idea that cell type differences can affect the mechanism of cytotoxicity.

A critical finding of this study is the identification of several genes that are regulated in response to treatment with the VEGF_121_/rGel fusion construct. We observed an increase in the RNA levels of several genes that are involved in inflammation, chemotaxis, intermediary metabolism, and apoptotic pathways (Table 1). To our knowledge, this microarray analysis is the first to be performed on cells treated with a gelonin-based therapeutic. A previous report showed that only two of these genes, MKP-1 and CXCR4, were also upregulated in HUVECs after treatment with VEGF_165 _for 24 h [17]. The present study shows that VEGF_121_/rGel is a member of the class of molecules that can prevent E-selectin-mediated metastasis because protein levels barely doubled in both PAE/VEGFR-2 and HUVECs after treatment with VEGF_121_/rGel. We observed a similar pattern of induction of RNA but not protein levels with other genes as well. Several genes involved in the control of the apoptotic pathway were modulated in response to the fusion toxin even though the overall cytotoxic effect on target cells did not include an observable impact on the apoptotic pathway. Taken together, we conclude VEGF_121_/rGel induces an increase in mRNA levels of genes that are important in cell adhesion, migration, and inflammatory response but generally does not induce a concomitant increase in protein expression. Since the rGel component of the fusion construct operates by inhibiting protein synthesis, VEGF_121_/rGel could inhibit synthesis of critical proteins that are important for suppression of these specific genes. In our laboratory, current studies are under way in breast and prostate orthotopic and metastatic (i.e., lung and bone) tumor models to further characterize the effects of this drug *in vitro *and *in vivo*.

## Conclusions

Our study shows that the specific cytotoxic effect of VEGF_121_/rGel observed against tumor vasculature *in vivo *is due to targeting of endothelial cells that overexpress VEGFR-2. VEGF_121_/rGel is rapidly internalized into log-phase endothelial cells via VEGFR-2 and mediates a robust cytotoxic effect that is primarily necrotic and negates the upregulation of genes involved in inflammation, chemotaxis and transcription regulation. However, modulation of these genes may influence tumor development in addition to exerting direct cytotoxic effects on the tumor neovasculature. Therefore, important considerations for future study are the effects of VEGF_121_/rGel cytotoxicity on tumor endothelial cells and the potential bystander effects of the construct on adjacent cells in the tumor microenvironment.

## Competing interests

The authors declare that they have no competing interests.

## Authors' contributions

KAM carried out the purification of VEGF_121_/rGel, performed the in vitro studies and drafted the manuscript. SR performed the cell surface binding of radiolabeled VEGF_121_/rGel, and helped draft the manuscript. CG-M performed the CAM assay, interpreted the results and helped draft the manuscript. LR performed the statistical analysis of the microarray data and helped draft the manuscript. JX performed the CAM assay and participated in data collection and interpretation. SK prepared, induced and harvested E. coli cells that expressed VEGF_121_/rGel. LHC participated in the design of the VEGF_121_/rGel construct. WNH performed the confocal microscopy and helped draft the manuscript. WZ performed the microarray analysis experiment and coordinated interpretation of the microarray data. JW provided the PAE/VEGFR-1 and PAE/VEGFR-2 cells and participated in the design of the study. PET participated in the design of the study. MGR participated in the design of the study and helped draft the manuscript. All authors read and approved the final manuscript.

## Pre-publication history

The pre-publication history for this paper can be accessed here:

http://www.biomedcentral.com/1471-2407/11/358/prepub

## Supplementary Material

Additional File 1 **At an equivalent molar concentration as VEGF_121_/rGel (48 nM), no internalization of recombinant gelonin (rGel) was detected in PAE/VEGFR-2 or PAE/VEGFR-1 cells over 24 h**. Click here for file

Additional File 2 **As with PAE/VEGFR-2 cells, cytotoxicity of VEGF_121_/rGel on PAE/VEGFR-1 cells is dependent on exposure time, but overall cytotoxicity is significantly lower. rGel exposure time is 72 h**. Click here for file

Additional File 3 **Treatment of PAE/VEGFR-2 cells with VEGF_121 _or VEGF_121_/rGel does not result in PARP cleavage**. Click here for file

Additional File 4 **Upregulation of E-Selectin in HUVECs over 24 h after treatment with VEGF_121_/rGel, as determined by PCR analysis. GAPDH was used as a control for loading**. Click here for file

Additional File 5 **MKP-1 protein expression did not correspond to a 2.7-fold increase in RNA levels in HUVECs over 24 h**. ERK-2 protein expression, previously shown to be regulated by MKP-1 in HUVECs, also showed no change in protein expression. Click here for file
