# Supplementary material for: Cytotoxicity of VEGF121/rGel on vascular endothelial cells resulting in inhibition of angiogenesis is mediated via VEGFR-2
Source: BMC Cancer. 2011 Aug 17;11:358. doi: 10.1186/1471-2407-11-358 (PMC3176242; doi:10.1186/1471-2407-11-358)
Supplement: Additional File 3 — Treatment of PAE/VEGFR-2 cells with VEGF121 or VEGF121/rGel does not result in PARP cleavage. [file 1471-2407-11-358-S3.DOC]

**Cytotoxicity of VEGF_121_/rGel on Vascular Endothelial Cells Resulting in Inhibition of Angiogenesis is Mediated via VEGFR-2**

Khalid A. Mohamedali, Sophia Ran, Candelaria Gomez-Manzano, Latha Ramdas, Jing Xu, Sehoon Kim, Lawrence H. Cheung, Walter N. Hittelman, Wei Zhang, Johannes Waltenberger, Philip E. Thorpe, and Michael G. Rosenblum

**Additional File 3**

Treatment of PAE/VEGFR-2 cells with VEGF_121_ or VEGF_121_/rGel does not result in PARP cleavage.
