# Supplementary material for: Cytotoxicity of VEGF121/rGel on vascular endothelial cells resulting in inhibition of angiogenesis is mediated via VEGFR-2
Source: BMC Cancer. 2011 Aug 17;11:358. doi: 10.1186/1471-2407-11-358 (PMC3176242; doi:10.1186/1471-2407-11-358)
Supplement: Additional File 4 — Upregulation of E-Selectin in HUVECs over 24 h after treatment with VEGF121/rGel, as determined by PCR analysis. GAPDH was used as a control for loading. [file 1471-2407-11-358-S4.DOC]

**Cytotoxicity of VEGF_121_/rGel on Vascular Endothelial Cells Resulting in Inhibition of Angiogenesis is Mediated via VEGFR-2**

Khalid A. Mohamedali, Sophia Ran, Candelaria Gomez-Manzano, Latha Ramdas, Jing Xu, Sehoon Kim, Lawrence H. Cheung, Walter N. Hittelman, Wei Zhang, Johannes Waltenberger, Philip E. Thorpe, and Michael G. Rosenblum

**Additional File 4**

Upregulation of E-Selectin in HUVECs over 24 h after treatment with VEGF_121_/rGel, as determined by PCR analysis. GAPDH was used as a control for loading.
