# Supplementary material for: Cytotoxicity of VEGF121/rGel on vascular endothelial cells resulting in inhibition of angiogenesis is mediated via VEGFR-2
Source: BMC Cancer. 2011 Aug 17;11:358. doi: 10.1186/1471-2407-11-358 (PMC3176242; doi:10.1186/1471-2407-11-358)
Supplement: Additional File 5 — MKP-1 protein expression did not correspond to a 2.7-fold increase in RNA levels in HUVECs over 24 h. ERK-2 protein expression, previously shown to be regulated by MKP-1 in HUVECs, also showed no change in protein expression. [file 1471-2407-11-358-S5.DOC]

**Cytotoxicity of VEGF_121_/rGel on Vascular Endothelial Cells Resulting in Inhibition of Angiogenesis is Mediated via VEGFR-2**

Khalid A. Mohamedali, Sophia Ran, Candelaria Gomez-Manzano, Latha Ramdas, Jing Xu, Sehoon Kim, Lawrence H. Cheung, Walter N. Hittelman, Wei Zhang, Johannes Waltenberger, Philip E. Thorpe, and Michael G. Rosenblum

**Additional File 5**

MKP-1 protein expression did not correspond to a 2.7-fold increase in RNA levels in HUVECs over 24 h. ERK-2 protein expression, previously shown to be regulated by MKP-1 in HUVECs, also showed no change in protein expression.
